# Exploratory Studies on RNAi-Based Therapies Targeting Angiotensinogen in Hypertension: Scoping Review

**DOI:** 10.3390/jpm15010003

**Published:** 2024-12-25

**Authors:** Antonio da Silva Menezes Junior, Thallys Henrique Marques Nogueira, Khissya Beatryz Alves de Lima, Henrique Lima de Oliveira, Silvia Marçal Botelho

**Affiliations:** 1 Medicine School, Pontifical Catholic University of Goiás, Goiânia 74605-010, Brazil; 20202006000426@pucgo.edu.br (T.H.M.N.); khissya_beatryz@discente.ufg.br (K.B.A.d.L.); silviamarcal@ufg.br (S.M.B.); 2 Faculty of Medicine, Internal Department, Federal University of Goiás, Goiânia 74001-970, Brazil; henique.lima2@discente.ufg.br

**Keywords:** angiotensinogen, antisense oligonucleotides, hypertension, RNAi-based therapies, RNA interference, small interfering RNA

## Abstract

**Background:** Systemic arterial hypertension contributes to cardiovascular morbidity and mortality worldwide. Many patients cannot achieve optimal blood pressure (BP) control with traditional therapies, which often results in poor patient adherence and limited long-term efficacy. We investigated the potential of RNA interference (RNAi) therapies targeting hepatic angiotensinogen (AGT) for hypertension management. **Methods:** This scoping review was conducted by the Joanna Briggs Institute, following a six-stage methodological framework and adhering to PRISMA recommendations. A comprehensive search was conducted across seven databases to identify relevant studies published until May 2024. Data extraction was performed separately, and both quantitative and qualitative analyses were conducted. A population, concept, and context model-based search was performed, selecting controlled MeSH terms and uncontrolled descriptors and cross-referencing them using Booleans. **Results:** Fifteen articles met our inclusion criteria. Focusing on the efficacy and safety of RNAi-based therapies, this review discusses several key approaches, including antisense oligonucleotides (IONIS-AGT-LRx), small interfering RNA (siRNAs; zilebesiran), and adeno-associated viruses carrying short hairpin RNAs. Notably, zilebesiran conjugated with N-acetylgalactosamine significantly reduced systolic BP by 20 mmHg, sustained for up to six months post-administration, with minimal adverse effects. **Conclusions:** RNAi-based therapies, particularly those using siRNAs, such as zilebesiran, are promising for the treatment of hypertension. They offer long-term BP control with fewer doses, potentially improving patient adherence and outcome. Although these therapies address several limitations of current antihypertensive treatments, further studies are required to confirm their long-term safety and efficacy.

## 1. Introduction

Systemic arterial hypertension (SAH) is a significant global public health challenge and one of the primary risk factors for cardiovascular diseases worldwide [1]. The number of hypertensive patients doubled from 1990 to 2019, reaching 1.28 billion, and studies from the Global Burden of Disease (GBD) project that SAH causes 10.8 million annual deaths and an overall burden of 235 million disability-adjusted life years [2,3]. Despite these consequences, less than half of the hypertensive population on treatment has controlled blood pressure (BP), with only 23% of women and 18% of men achieving the ideal BP range [4,5].

One of the main difficulties in effectively controlling SAH is poor adherence to medications, which is influenced by the number of pills required daily and the variety of adverse effects these medications may cause throughout the life of an asymptomatic condition [6,7]. Furthermore, existing therapies for BP reduction can lose effectiveness over time due to the renin–angiotensin–aldosterone system (RAAS) escape phenomenon, which, through compensatory pathways, causes an increase in renin and the restoration of angiotensin II (Ang II). Other pathways related to hypertension mechanisms are oxidative stress, endothelial dysfunction, and sympathetic nervous system overactivation, which contribute to hypertension. While not the focus of this review, these pathways are critical to understanding the multifactorial nature of hypertension [8,9].

The RAAS plays a crucial role in regulating BP, and its dysfunction is closely related to the development and progression of hypertension. The primary effector of RAAS is Ang II, which results from angiotensin I (Ang I) cleavage by angiotensin-converting enzyme (ACE). Ang I results from the renin-mediated cleavage of angiotensinogen (AGT) produced in the liver [2]. Preclinical and clinical evidence has recently suggested that antihypertensive drugs based on silencing hepatic AGT production through RNA interference (RNAi) could improve adherence to hypertension treatment [8,9].

Therapeutic approaches used to silence hepatic AGT include antisense oligonucleotides (ASOs), small interfering RNAs (siRNAs), and adeno-associated viruses carrying short hairpin RNA (AAV-shRNA), with the latter being restricted to animal model experiments [2,10]. The key difference between these techniques is that ASOs are single-stranded (ss) molecules; whereas, siRNAs are double-stranded (ds) [11]. Double-stranded RNA (dsRNA) is more specific and easier to use than ssRNA for silencing. siRNAs delivered through lipid nanoparticles were the first to demonstrate the efficacy of RNAi in humans [2,10,11].

This review evaluates new RNAi therapeutic approaches for SAH targeting hepatic AGT, highlighting existing knowledge gaps and identifying opportunities for future research on hypertension and emerging gene silencing therapies.

## 2. Methods

### 2.1. Study Type, Protocol, and Registration

This scoping review aimed to evaluate new RNA-based therapeutic approaches for hypertension. We focused on documenting, gathering, and synthesizing information on the silencing of hepatic AGT by RNAi to improve the effectiveness of SAH treatment and reduce morbidity and mortality. Additionally, we sought to identify existing knowledge gaps in scientific research and to provide an instrument to aid clinical decision making.

This type of study requires an interactive process conducted in five stages: (1) the identification of the research question; (2) the identification of relevant studies; (3) the selection of studies; (4) data mapping; and (5) grouping, analyzing, and summarizing data [12]. Moreover, this study was based on the stages proposed by the Preferred Reporting Items for Systematic Reviews and Meta-Analyses Extension for Scoping Reviews (PRISMA-ScR). The final protocol was registered in the Open Science Framework (OSF) on December 2024, register DOI: https://doi.org/10.17605/OSF.IO/KZ6P4.

### 2.2. Review Question

The review question used the population, concept, and context (PCC) strategy. Hypertensive individuals formed the population; whereas, RNAi-based therapeutic approaches for silencing hepatic AGT and effective BP control constituted the concept and context, respectively. Thus, the final review question was: will new RNAi-based approaches targeting hepatic AGT effectively control BP?

### 2.3. Eligibility Criteria

Inclusion criteria: This review, appreciating the diverse nature of scientific research, included any articles that answered the research question. It covered studies published in any language, including reviews, clinical studies, experimental studies, and randomized controlled trials. There were no limitations regarding publication dates. However, secondary sources were excluded, such as editorials, books, expert opinion articles, theses, dissertations, and abstracts.

### 2.4. Information Sources and Study Selection

The following databases were searched: the Excerpta Medica Database (EMBASE), PubMed, Scopus, and Cochrane. After collecting articles according to the inclusion and exclusion criteria, the studies were imported into Rayyan software, and duplicates were removed. The first screening was based on the title and, in some cases, the abstract and full article. The selected articles were fully read and organized using an article filing tool.

Since there were no ethical or moral implications, and the content was available in online databases without participant identification information; submission to the Ethics Committee was unnecessary.

### 2.5. Search Strategy

A search strategy based on the PCC model was created by selecting controlled MeSH terms and uncontrolled descriptors and cross-referencing them using the following Booleans: “arterial hypertension” AND “RNA” AND “angiotensinogen”. PRISMA-ScR, an extension of PRISMA, was also used to support adherence to best practices in scoping the project design for publication [12].

### 2.6. Data Collection Process

After selecting the studies, the references of the selected articles were checked to expand the search and to verify whether any relevant studies had been excluded. The extracted data were rigorously analyzed and collected, filling out a characterization table in Microsoft Word for the following details: author/year of study, country where the study was conducted, study title (identification), type of study, study objective, methodology, and main findings.

### 2.7. Risk of Bias Assessment or Quality Assessment

As this scoping review was conducted to identify knowledge gaps, there was no risk of bias or quality assessment, according to the Joanna Briggs Institute (JBI) manual.

### 2.8. Data Synthesis

This review provides a qualitative synthesis of the data from selected studies, describing promising RNAi therapeutic approaches that silence hepatic AGT production and efficiently reduce BP.

## 3. Results

**Figure 1:**
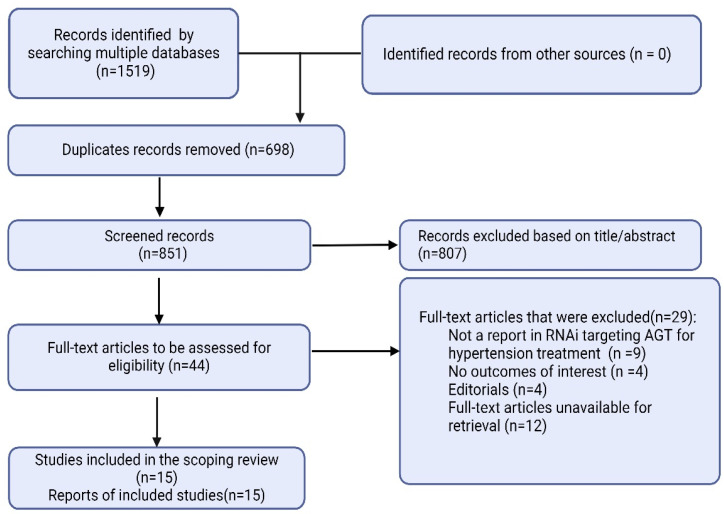
PRISMA flow diagram showing the different phases of this scoping review. AGT, angiotensinogen; RNAi, RNA interference.

A total of 1519 articles were found, of which 698 were duplicates, and 851 were unrelated to AGT RNAi. Among the 44 articles selected for full reading, nine were not focused on RNAi targeting AGT for hypertension treatment, 29 were not fully available, and 12 were unavailable for retrieval. Thus, 15 articles were selected for the final analysis (Figure 1). The primary data for each study are presented in Table 1.

The analysis categories established from the comprehensive and critical reading of each article were as follows: (A) role of angiotensinogen in cardiovascular diseases, (B) mechanism of action of RNA-based therapies for hypertension treatment, (C) preclinical studies of RNA-based therapies for hypertension, and (D) human studies of RNA-based therapies for hypertension.

The analysis categories were established after carefully reading the articles and observing the main discussion topics. Therefore, five subdivisions were created to extract the main subjects and discuss new therapeutic possibilities for treating SAH.

### 3.1. The Role of AGT in Cardiovascular Diseases

**Figure 2:**
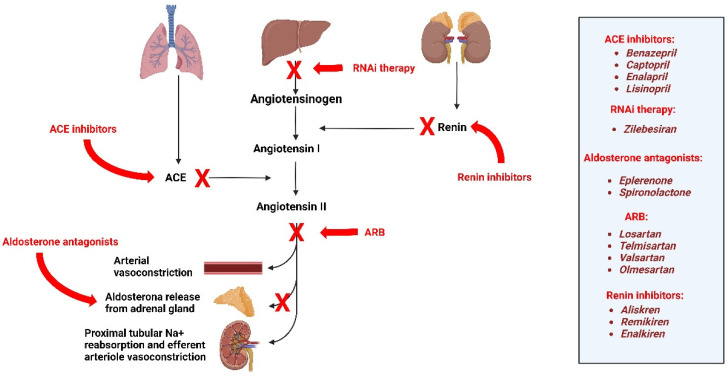
The renin–angiotensin–aldosterone system (RAAS). Legends: ACE, angiotensin-converting enzyme. ARB, angiotensin receptor blocker; RNAi, RNA interference.

RAAS produces the precursor protein AGT in the liver and smaller amounts in the kidneys, heart, brain, adipose tissue, and blood vessels [18,22]. AGT is cleaved into Ang I through the action of renin, a protein released from the juxtaglomerular cells lining the afferent arteriole of the kidney. Ang I is then cleaved into Ang II by ACE, primarily found in the lungs. Ang II promotes arterial vasoconstriction by contracting smooth muscle [1], stimulates aldosterone release in the glomerulosa zone of the adrenal gland [2], increases sodium reabsorption in the proximal tubule of the kidneys [3], and leads to efferent arteriolar vasoconstriction, causing glomerular capillary hypertension [4]. Consequently, when positively regulated, AGT increases BP by altering the vascular tone, blood volume, electrolyte balance, and aldosterone synthesis, causing tissue remodeling and target organ damage [14,22] (Figure 2).

Consequently, although the regulation and functioning of local or tissue RAAS are important in maintaining normal cardiac performance, they can also contribute to the development of cardiovascular diseases [24,25]. Therefore, innovative RNA-based techniques targeting specific organs could provide new therapeutic possibilities and tools to improve our understanding of cardiovascular diseases [15].

Animal model studies have reported that rats or mice carrying the human or rat *AGT* gene exhibit cardiac hypertrophic phenotypes and increased B-type natriuretic peptide (BNP) levels [26,27], and other mechanisms should be associated with genetic expression as oxidative stress and inflammatory pathways. Conversely, mice with global or cell-specific AGT deficiency show reduced BP. RNA therapies that target hepatocytes to inhibit hepatic AGT in rats and nonhuman primates have demonstrated that extrahepatic AGT depends on hepatic AGT synthesis [23,28]. These findings underscore the importance of directly silencing RNA-based therapies targeting hepatic AGT, specifically, avoiding systemic off-target effects, as explored in the present study.

Additionally, data on extrahepatic AGT production are sometimes conflicting, as AGT depletion in adipocytes either reduces or does not affect plasma AGT levels [8]. AGT is highly expressed in perivascular adipose tissue surrounding vessels, and selective AGT depletion in brown adipose tissue can decrease BP. Therefore, targeting these two types of adipose tissues in the liver may result in successful new therapies, as supported by the current literature [25].

*AGT* gene–environment interactions should also be discussed, as they play important roles in the pathophysiology of cardiovascular diseases, such as heart failure and hypertension [15,29]. An experimental study in mice revealed the existence of two AGT polymorphisms: haplotype I (hap-I), known as the +1164A variant, and haplotype II (hap-II), known as the +1164G variant. Animals with transgenic hap-I chromatin showed stronger binding to glucocorticoid receptor transcription factors and hepatocyte nuclear factor 3β than hap-II animals, resulting in higher plasma AGT concentrations and the development of hypertension [8,15].

In recent decades, these findings have led to an increase in studies on the development of AGT-targeted therapies. Approaches to manipulate AGT expression in rodents, such as those involving ASOs, siRNAs, and AAV-shRNAs, have demonstrated efficiency in suppressing plasma AGT concentration and reducing BP. These approaches range from cell-specific mouse genetic manipulation models to RNA-based therapies targeting specific organs [10,15,30].

### 3.2. Mechanism of Action of RNA-Based Therapies for Hypertension Treatment

Recently, noncoding RNAs responsible for RNAi have been shown to regulate protein expression and have high therapeutic potential. RNAi therapies can use three strategies: ASO, siRNA, and shRNA. shRNA is a precursor of siRNA, consisting of dsRNA with a hairpin loop on one side [6,31].

Both ASOs and siRNAs bind to the target RNA through Watson–Crick base pairing, despite having different mechanisms of action. Generally, protein production begins in the nucleus with the transcription of a gene into pre-messenger RNA (pre-mRNA), which immediately undergoes nucleotide modifications to form mature mRNA. The molecule is then transported to the cytoplasm, where it is translated by ribosomes. ASOs are 8–50 nucleotide ssRNA molecules designed with an antisense orientation to that of their target mRNAs. ASOs bind to target mRNAs in the cytoplasm or nucleus upon cell entry via endocytosis. Enzymatic cleavage by RNase H destroys the target mRNA [2,18,23].

Similarly, synthetic siRNAs composed of 20–24 base pairs of dsRNA enter the cell via endocytosis. Upon escape from the endosome, they bind to the RNA-induced silencing complex (RISC) containing a functional nuclease core. The antisense strand is recognized as the guide strand, forming a mature RISC and degrading the nonguide strand. The RISC complex binds to the target mRNA and silences protein synthesis [2,32]. shRNAs are small RNAs of approximately 80 nucleotides that feature internal hybridization, creating a short hairpin structure. They are introduced into cells via bacterial or viral vectors and converted into siRNAs, following the same process used to degrade the target mRNA. shRNAs can overcome the difficulty of transfecting certain cell types due to the possibility of using viral vectors for delivery [17,18,21].

Comparatively, ASOs enter cells more easily than dsRNAs but require more frequent administration and have higher pharmacological tolerance [2,33,34]. Interestingly, siRNAs are more stable and require less frequent application, because the RISC complex is recycled after cleaving the target mRNA. The same guide strand is reused for multiple rounds of target mRNA cleavage. Recent studies have shown that siRNAs can be stored in liver endosomes, creating a depot [35,36].

In in vivo gene silencing, RNAi-based drug delivery across the membrane is the main challenge faced by the pharmaceutical industry, because the lipid bilayer is adapted to protect the cell against large, hydrophilic, and negatively charged molecules. Since ASOs and siRNAs are macromolecules, endocytosis traps them in endosomes [2,18]. Notably, the development of lipid nanoparticles (LNPs) has significantly improved RNAi delivery in humans, overcoming dose-related toxicity and suboptimal efficacy problems [18,33]. Olearczyk et al. [18] showed that LNP-encapsulated siRNAs can modulate hepatic AGT mRNA expression and plasma protein levels, indicating AGT mRNA’s potent and specific silencing without affecting liver function.

Despite these advances, AGT-targeting ASO and siRNA therapies administered subcutaneously and intravenously require weekly doses and have safety profiles below expectations, including those involving LNP-encapsulated siRNA [37]. Targeted delivery to the liver is justified, because systemic AGT production depends on hepatic AGT levels. Furthermore, the liver contains fenestrated endothelial capillaries, which make it more receptive to systemically delivered drugs. Hepatocytes can be targeted by conjugating RNA-based drugs with trivalent N-acetylgalactosamine (GalNAc), allowing a single dose to sustain long-term effects [2,34,38].

The asialoglycoprotein receptor (ASGPR), also known as the Ashwell–Morell receptor, is expressed in hepatocytes and facilitates both the uptake and clearance of circulating glycoproteins with exposed terminal galactose residues and GalNAc glycans via endocytosis. Consequently, the GalNAc-siRNA architecture allows for highly efficient uptake by hepatocytes through interactions with ASGPR, followed by cellular internalization of the receptor and ligand with the siRNA cargo [17].

In this context, several RNAi therapy-based drugs for treating a variety of disorders, from rare diseases to common conditions, have emerged, including the following FDA-approved GalNAc-siRNAs: givosiran (for acute hepatic porphyria), lumasiran (for primary hyperoxaluria), inclisiran (for hypercholesterolemia), and vutrisiran (for hereditary transthyretin-mediated amyloidosis with polyneuropathy) [39].

Additional chemical modifications were made to first-generation GalNAc-siRNA conjugates to improve hepatic AGT mRNA silencing at lower doses [8]. A previous report emphasized that these chemical modifications allow for prolonged stability in the intracellular compartments of hepatocytes, generating continuous functional RNA-induced silencing complexes and sustaining RNAi activity for extended periods [40]. Additionally, a recent modification has increased the clinical safety of GalNAc-siRNA conjugates by reducing their potential for off-target mRNA binding [17]. Although GalNAc-ASO conjugation also improves drug delivery to hepatocytes, it has a poorer safety profile than GalNAc-siRNA and requires weekly subcutaneous doses, including their mechanism in inhibiting the conversion of Ang I to Ang II and their limitations due to RAAS escape.

### 3.3. Preclinical Studies of RNA-Based Therapies for Hypertension

Over the past two decades, genetic silencing methodologies have been used to silence RAAS genes in preclinical models of spontaneously hypertensive rats (SHR), demonstrating that ASOs targeting AT1 receptor genes, AGT, angiotensin-converting enzymes, and β1-adrenergic receptors effectively reduce BP. Table 2 shows the results of the most relevant preclinical trials. However, targeting AGT is the best approach, because conventional small-molecule inhibition is unsuccessful [17,18].

Initially, genetic silencing therapies achieved a maximum reduction of 50% in circulating AGT levels after a one-week treatment. In addition, DOCA, a mineralocorticoid receptor agonist, has been used in various models and has proven helpful in understanding how brain angiotensin generation depends on hepatic AGT. DOCA-complemented treatments also reduced cardiac hypertrophy, suggesting that siRNAs can block Ang II formation at cardiac tissue sites [34,41,42,43].

In a 5/6 nephrectomized Sprague Dawley rat model, BP increased slowly, and GalNAc-siRNA AGT treatment prevented further increases, highlighting a low-renin model offering renoprotection by suppressing renal Ang II. Notably, in a diabetic TGR (mRen2)27 rat model, increased BP is due to renin overexpression and is not directly related to diabetes; renin overexpression also offers renoprotection [34].

**Table 2:** Animal models treated with RNA-based therapies.

Drug	Class	Animal Model	Results	References
GPE-AGT-shRNA	siRNA	Spontaneously hypertensive rats	Reduced BP and decreased LVW/BW and HW/BW ratios	[17]
r-AAV-AGT-ASO	ASO	Rats	Reduced plasma AGT concentration and decreased BP	[44]
r-AAV-AGT-ASO	ASO	Rats (intracardiac injection)	Reduced hepatic AGT concentration and decreased BP	[45]
GalNAc-AGT-siRNA	siRNA	Rats (subcutaneous injection)	Reduced plasma AGT concentration, decreased BP, and HW/BW ratio	[9,10]
Gen-AGT-ASO	ASO	Mice (intraperitoneal injection)	Reduced plasma AGT concentration and decreased BP	[10]
GalNAc-AGT-ASO	ASO	Mice (subcutaneous injection)	Reduced plasma and hepatic AGT concentration and decreased BP	[10]

AGT, angiotensinogen; ASO, antisense oligonucleotide; BP, blood pressure; BW, body weight; GalNAc-AGT-ASO, N-acetylgalactosamine-angiotensinogen-antisense oligonucleotide; GalNAc-AGT-siRNA, N-acetylgalactosamine-angiotensinogen-small interfering RNA; GPE-AGT-shRNA, gene promoter element-angiotensinogen-short hairpin RNA; HW, heart weight; LVW, left ventricular weight; r-AAV-AGT-ASO, recombinant adeno-associated virus-angiotensinogen-antisense oligonucleotide; siRNA, small interfering RNA.

### 3.4. Human Studies of RNA-Based Therapies for Hypertension

Two RNA-based therapeutics capable of silencing hepatic AGT, IONIS-AGT-LRx and ALN-AGT, are currently being tested to treat hypertension. The corresponding clinical trials are summarized in Table 3.

#### 3.4.1. IONIS-AGT-LRx

**Figure 3:**
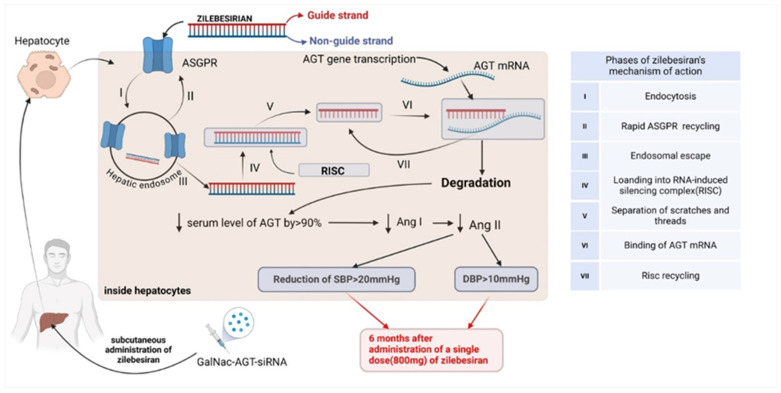
Mechanism of action of ASO therapies. AGT, angiotensinogen; Ang I, angiotensin I; Ang II, angiotensin II; ASO, antisense oligonucleotide; ASPGR, asialoglycoprotein receptor; AT1, angiotensin II type 1 receptor; GalNAc, N-acetylgalactosamine; mRNA, messenger RNA.

IONIS-AGT-LRx is an ASO conjugated to GalNAc, which targets hepatic *AGT* mRNA. Preliminary human study data have shown promising results for reducing AGT levels and improving treatment safety. ACE inhibitors reduce Ang II levels by approximately 60–70%, while RNA-based therapies, such as zilebesiran, achieve a >90% reduction in AGT levels. A phase 1 study with six ascending single-dose cohorts (5, 10, 20, 40, 60, or 80 mg; n = 29) and a placebo group (n = 12), as well as two ascending multiple-dose cohorts (40 and 80 mg; n = 16) and a placebo group (n = 4), in which the treatment was administered weekly for six weeks to healthy individuals, showed a 60% reduction in the plasma AGT levels of members of the single-dose 80 mg group, which returned to baseline by week ten without affecting BP or Ang II levels [2], emphasizing the recycling of the RISC complex, which ensures prolonged activity, as seen in Figure 3.

A pioneering phase 2 trial was randomized for the application of IONIS-AGT-LRx (n = 17) or placebo (n = 8) in 25 patients with controlled hypertension on two antihypertensive medications (ACE inhibitors or angiotensin receptor blockers with beta-blockers, calcium channel blockers, or diuretics), as we have reinforced in the introduction section. Patients receiving 80 mg weekly doses of IONIS-AGT-LRx by subcutaneous injection (seven doses) showed a 53% reduction in AGT levels by day 43. This reduction was significant from day 8 to day 78, suggesting the durability of the effect despite weekly application. The primary outcomes were not BP effects; however, an 8 mm Hg reduction in systolic BP with no decrease in Ang II or increase in renin was noted.

Another phase 2 study with 26 participants with treatment-resistant hypertension receiving 2–3 antihypertensive medications showed a significant 67% AGT reduction by day 57 in those receiving 80 mg weekly IONIS-AGT-LRx doses (nine doses in total). However, no significant reduction in BP was observed, as shown in Table 4.

No acute kidney injury or hyperkalemia was observed in animal studies following AGT silencing. More extensive studies at doses that reduce BP are needed to include ASO-based medications in clinical practice and evaluate their safety and tolerability in more representative groups (elderly patients and patients with chronic kidney disease, chronic heart failure, and/or type 2 diabetes). The ASTRAAS (NCT04714320) phase 2 randomized clinical trial, which concluded in February 2023, included up to 150 patients with hypertension, with the primary outcome being a change in BP; however, the results are yet to be published.

#### 3.4.2. ALN-AGT (Zilebesiran)

**Figure 4:**
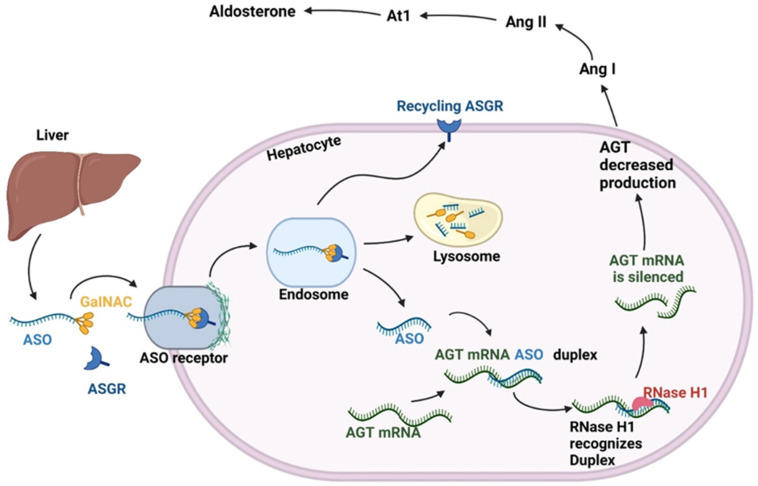
Mechanism of action of zilebesiran. AGT, angiotensinogen; Ang I, angiotensin I; Ang II, angiotensin II; ASGPR, asialoglycoprotein receptor; DBP, diastolic blood pressure; GalNAc, N-acetylgalactosamine; mRNA, messenger RNA; RISC, RNA-induced silencing complex; SBP, systolic blood pressure; siRNA, small interfering RNA.

Zilebesiran is the designated name for an experimental GalNAc-siRNA therapeutic that targets human hepatic AGT mRNA and eventually inhibits AGT protein production (Figure 4). A phase 1 randomized, double-blind, placebo-controlled clinical trial evaluated the pharmacokinetics, pharmacodynamics, clinical safety, and impact of zilebesiran on BP [8].

Patients received either ascending single subcutaneous doses of zilebesiran (10–800 mg) or placebo. Zilebesiran was correlated with dose-dependent reductions in serum AGT levels. Additionally, single doses of zilebesiran (≥200 mg) resulted in systolic and diastolic BP reductions (>ten mmHg and >5 mmHg, respectively) by week 8, which was consistent throughout the 24 h diurnal cycle and was sustained for up to 24 weeks. Patients receiving 800 mg of zilebesiran under high-sodium diet conditions had attenuated BP reductions. In contrast, co-administration with irbesartan resulted in incremental decreases in SBP and DBP (−63 ± 31 mmHg and −3 ± 19 mmHg, respectively). Phase 1 results strongly support the RNAi-based approaches targeting hepatic AGT as an effective human BP reduction strategy (20–25 mmHg). Single doses of up to 800 mg were well tolerated without hypotension, hyperkalemia, or worsening renal function. Zilebesiran demonstrated a prolonged pharmacodynamic profile with sustained AGT serum reduction and BP lowering for up to 24 weeks [8].

A phase 2 KARDIA-1 trial (NCT04936035), a randomized, double-blind, multicenter, placebo-controlled study, evaluated the efficacy and safety of zilebesiran in patients with mild-to-moderate hypertension, focusing on its effects. The results supported quarterly or semiannual subcutaneous zilebesiran dosing, achieving consistent pharmacodynamic effects and effective BP reduction for six months (average 10–15 mmHg reduction) with RNA therapies achieving sustained reductions of up to 25 mmHg. BP reduction with a single medication dose persisted until month 6, particularly at 300 and 600 mg doses. Mild-to-moderate adverse events, such as injection site reactions and hyperkalemia, have been reported. These efficacy, safety, and pharmacodynamic effects extend phase 1 findings for zilebesiran [16].

In an ongoing phase 2 KARDIA-2 study (NCT05103332), the efficacy and safety of zilebesiran as an adjunct therapy in patients with inadequately controlled hypertension on standard antihypertensive medications are evaluated. This study is expected to be completed by 2025.

Less frequent dosing of zilebesiran may enhance treatment adherence compared to that of currently available antihypertensive drugs. Moreover, unlike the variable RAAS blockade from daily dose drugs, it offers a continuous and durable one. A series of clinically approved products and substantial preclinical studies have established that GalNAc-siRNA conjugates targeting the liver have favorable preclinical safety profiles and broad therapeutic windows [8].

The KARDIA-1 phase 2 trial included 394 patients who received subcutaneous doses of zilebesiran (150 mg, 300 mg, or 600 mg) administered quarterly (Q3M) or biannually (Q6M). Significant reductions in 24-h mean systolic blood pressure (SBP) were observed across all dosing regimens. At month 3, placebo-adjusted SBP reductions were 14.1 mmHg (150 mg Q6M), 16.7 mmHg (300 mg Q6M), and 15.7 mmHg (600 mg Q6M) (*p* < 0.0001 for all). These reductions were sustained through month 6, with decreases ranging from 11.1 mmHg (150 mg Q6M) to 14.5 mmHg (300 mg Q6M), indicating durable efficacy. Office SBP measurements aligned with these findings, reinforcing the consistency of zilebesiran’s antihypertensive effects over time. Such sustained BP control, achieved with only two or four annual doses, marks a significant advancement over traditional antihypertensive therapies requiring daily administration [46].

In addition to its efficacy, zilebesiran demonstrated a favorable safety and tolerability profile. Adverse events (AEs) were comparable between zilebesiran and placebo groups, with common AEs including injection site reactions, hyperkalemia, and upper respiratory tract infections. Serious adverse events (SAEs) occurred in 3.6% of zilebesiran-treated patients, compared to 6.7% in the placebo group, with no SAEs attributed to the study drug. Importantly, the study highlighted zilebesiran’s potential to address the key limitations of current antihypertensive therapies, such as poor adherence and variable efficacy, as shown in Table 4. The biannual dosing schedule simplifies treatment regimens and ensures sustained blood pressure reductions, mitigating the RAAS escape phenomenon often observed with ACE inhibitors and ARBs. These findings position zilebesiran as a promising therapeutic option for long-term hypertension management, warranting further investigation in larger and more diverse populations to confirm its safety and efficacy, particularly in patients with comorbid conditions [47,48,49].

Therapeutic AGT depletion using siRNA may impair RAAS activation, which is necessary to maintain adequate BP and tissue perfusion during shock and other hypotensive conditions. Therefore, the risk of refractory hypotension owing to direct adverse effects or impaired RAAS activation during hemodynamic stress caused by an unforeseen volume of depletion, bleeding, infection, or cardiac problems is an important concern. Consequently, therapeutic reversal agents for RNAi have been investigated.

## 4. Discussion

Health problems related to hypertension continue to affect millions of people despite the availability of many antihypertensive medications. Currently, only one in five patients with hypertension achieves ideal BP control, reflecting poor treatment adherence, incomplete detection, and the ineffectiveness of current drugs, which require repeated use and multiple doses and lead to numerous adverse reactions. BP is a significant risk factor, owing to its substantial impact on the structure and function of organs, such as the heart, brain, and kidneys, potentially leading to their failure. Therefore, the need for a new antihypertensive agent that synergizes with traditional antihypertensive drugs is essential for the treatment of refractory hypertension and cardiac hypertrophy [21].

This study provides valuable insights into RNA-based therapies for hypertension, highlights the importance of AGT in BP regulation, and discusses the efficacy of different therapeutic approaches that target hepatic AGT. ASO IONIS-AGT-LRx treatment reduced BP to a lesser extent than zilebesiran (siRNA), which showed a significant and sustained BP reduction up to six months after dosing. Moreover, zilebesiran conjugated with GalNAc allowed for targeted and efficient delivery to hepatocytes, resulting in prolonged and effective AGT knockdown. The safety of siRNAs in future therapeutic implementations could be supported by their ability to reverse the effects of conventional vasopressors in medical emergencies in which the RAAS is essential.

Several studies have confirmed the efficacy of siRNAs in reducing BP by comparing their results to those of other studies. Uijl et al. [10] and Yuan et al. [17] showed that AGT-targeted siRNA treatment results in sustained BP reduction in SHR. These results are consistent with Morgan et al. [50], indicating that siRNAs have superior efficacy than ASOs in reducing BP [10,17,21,50].

A review by Addison et al. [2] highlighted that, owing to their high specificity and durability, siRNA-based treatments offer significant advantages over other RNA-based therapies, such as those using ASOs, which require more frequent administration and have lower stability. This reinforces the idea that siRNA-based therapies may be a more effective solution for hypertension, particularly in patients with poor adherence to conventional treatments [2].

Estimates suggest that over half of hypertensive patients become partially or entirely nonadherent to treatment with currently available antihypertensives within a year of starting, resulting in adverse effects. Therefore, new medications with fewer adverse effects and higher efficacy should be developed to address poor adherence [2,14,22].

RNAi is a promising and innovative approach to hypertension treatment. IONIS-AGT-LRx and zilebesiran are significant advances in suppressing a unique RAAS precursor of AGT. Zilebesiran showed remarkable results in initial clinical trials, with phase 1 data indicating good tolerance and significantly sustained BP and plasma AGT levels. Its potential application every six months could improve treatment adherence. In contrast, IONIS-AGT-LRx did not considerably reduce BP. Additional phase 2 studies are necessary to evaluate their potential [2,14,23].

Another potential advantage of RNAi-based drugs is their ability to reduce BP for >24 h. Robust studies have shown an association between nondipper (0–10% night reduction) or riser (≤0%) BP patterns and increased cardiovascular risk. Thus, drugs such as ASOs or siRNAs targeting AGT may help regulate physiological nighttime BP drop patterns [19,46,47,48,49].

Future trials are needed to confirm the efficacy and safety of these therapies in populations with associated comorbidities, such as type 2 diabetes, chronic kidney disease, and heart failure. Additionally, preparing for emergency medical management in patients undergoing AGT siRNA therapy presents challenges, as withdrawal alone may not suffice for reversal [19].

Three phase 2 randomized clinical trials of zilebesiran are currently underway. KARDIA-1 (NCT04936035) is a multicenter study evaluating the efficacy and safety of monthly subcutaneous injections in patients with mild-to-moderate hypertension. The initial results indicated a consistent BP reduction of ≥15 mmHg compared with the placebo group. KARDIA-2 (NCT05103332) investigated zilebesiran as an adjunct therapy for treatment-resistant hypertension. Before randomization, participants received a baseline antihypertensive agent, zilebesiran, or placebo. KARDIA-3 (NCT06272487) aims to evaluate zilebesiran as an adjunct therapy in patients with high cardiovascular risk and inadequately controlled hypertension [46,47,48,49].

Initial studies and clinical trials have shown that RNA-based therapies can trigger immune responses. For example, RNAi recognition by the immune system can produce inflammatory cytokines that can have adverse effects [51]. In addition, the long duration of action of some RNAi drugs increases the risk of long-term adverse effects, the extent of which remains unclear [52].

Another essential point is genetic variability among individuals, which can influence the response to RNAi-based therapy. Genetic polymorphisms can affect gene silencing efficacy, resulting in varied therapeutic responses [53]. Moreover, comorbidities and concomitant medication use can interfere with the action of RNAi, further complicating treatment response predictions [54].

Socioeconomic factors must also be analyzed. The development and production of RNAi-based therapies are expensive, limiting patient access to innovative treatments. Furthermore, the regulatory and approval processes are rigorous, requiring extensive clinical trials that further increase the cost and time for these therapies to reach the market. When comparing with existing treatments, the initial data suggest that RNAi-based therapies like zilebesiran offer sustained blood pressure control with fewer doses. However, comparative studies are needed to determine cost-effectiveness, especially in settings with high healthcare expenditure sensitivity. Regarding the clinical and economic benefits, infrequent dosing, reduced healthcare visits for medication adjustments, and possibly fewer long-term complications from poor BP control may offset higher upfront costs. Taking into account global health considerations, given the variability in healthcare budgets worldwide, we have stressed the need for more studies to determine the feasibility of implementing such therapies in different economic contexts. Regarding future research, we recommend health economics studies to compare the cost–benefit ratio of RNAi therapies to conventional BP medications, considering efficacy, safety, and patient compliance [55].

### 4.1. Clinical Evidence on Aliskiren Combination Therapy

Combining aliskiren with other antihypertensives, like amlodipine, lowers blood pressure better than monotherapy. A meta-analysis found that aliskiren–amlodipine reduced systolic and diastolic blood pressures without increasing adverse events. The renal results show that aliskiren added to an angiotensin II receptor blocker (ARB) did not delay end-stage renal disease or reduce proteinuria in advanced chronic kidney disease patients. Regarding safety concerns, in type 2 diabetes patients, aliskiren in combination with ARBs or ACEIs increased the risk of hyperkalemia and hypotension, so the ALTITUDE trial was stopped early [56]. While aliskiren combination therapy may improve blood pressure control, its cost-effectiveness is uncertain. Adverse events requiring additional medical interventions may offset economic benefits. Aliskiren combinations are also less economically attractive due to the availability of generic, cheaper antihypertensive agents with proven efficacy and safety. Current guidelines do not recommend adding aliskiren to standard antihypertensive regimens, especially those with ARBs or ACEIs, due to mixed clinical outcomes and safety concerns. More long-term studies are needed to prove combination therapies’ clinical benefits and cost-effectiveness [57,58].

Although aliskiren co-administration is a promising method for addressing angiotensin escape, the evidence does not support its widespread use over more cost-effective and established treatments. Optimizing adherence to existing therapies and exploring alternative blood pressure control strategies may be more practical for healthcare systems with limited budgets.

### 4.2. Future Research Directions

Studies assessing the systemic and central effects of RNAi therapies on neuroinflammation, oxidative stress, and cognitive outcomes are critical for Alzheimer’s disease [13,20]. For diabetic nephropathy, trials should explore long-term renal outcomes, safety in populations with advanced kidney disease, and potential interactions with standard antihypertensive and glycemic control therapies. African Americans have lower renin levels and lower responses to ACE inhibitors and ARBs, as is well known. These differences are caused by salt sensitivity, low renin hypertension, and genetics [44].

### 4.3. AGT siRNA Treatment Reversibility in Preclinical Studies

AGT siRNA therapies show promise for restoring BP in emergencies, such as hypovolemia, hypotension due to hemorrhage or sepsis, and acute renal injury. A recent preclinical study on SHRs focused on reversing the BP-lowering effects of siRNA-induced AGT knockdown. SHRs on a low-salt diet received AGT siRNA (10 mg/kg) once a week for four weeks to reduce their BP, and their sensitivity to Ang II and norepinephrine were evaluated before and after AGT levels were decreased by the siRNA [45]. In the last two weeks of the study, a group of SHRs was randomly assigned to receive fludrocortisone, midodrine, or a high-salt diet. Telemetry was used to assess BP. AGT siRNA significantly decreased AGT levels by more than 99%, resulting in a corresponding 19 mm Hg decrease in mean arterial pressure (MAP). After reducing AGT using siRNAs, Ang II application led to a considerable increase in MAP, likely because the inhibition of the renin–angiotensin system made AT1 receptors more sensitive to Ang II. However, AGT siRNA administration did not significantly alter the effect of norepinephrine on MAP. Furthermore, fludrocortisone and a high-salt diet gradually returned MAP to its original levels; whereas, midodrine did not show the same effect. A high-salt diet initially increased MAP, which returned to normal levels over time [59]. Thus, the potential reversibility of AGT-siRNA therapy in preclinical settings is promising.

Additionally, a specific oligonucleotide known as REVERSIR^TM^ has been shown to reverse the suppression of gene activity caused by siRNAs in mice, restoring protein levels targeted by the siRNA within four days. This study suggests that vasopressors, such as norepinephrine and Ang II, currently used to treat circulatory shock, may be helpful for quickly reversing siRNA-induced AGT decreases in high-dependency scenarios. Although the intravenous administration of NaCl appears promising, thorough testing is required. In non-emergency scenarios, oral NaCl or fludrocortisone may temporarily provide an alternative for patients who need to suspend treatment [59,60,61,62,63,64].

### 4.4. Limitations

We have numerous limitations. Firstly, we address the initial absence of extensive data regarding long-term safety. The paper acknowledges the potential of RNAi-based therapies like zilebesiran but fails to adequately discuss the long-term safety concerns associated with these novel treatments, particularly in heterogeneous populations and those with comorbidities. Second, the heterogeneity of the included studies complicates the synthesis of results into coherent conclusions, as the scoping review encompasses a wide range of preclinical and clinical studies employing diverse methodologies. Thirdly, there is insufficient analysis of the cost-effectiveness. While RNA-based therapies offer innovative concepts, their elevated manufacturing and delivery expenses are merely referenced without a comprehensive evaluation, resulting in ambiguity regarding their feasibility for widespread clinical adoption. Fourthly, while the review emphasizes hepatic AGT as the primary therapeutic target, it provides limited information on other emerging targets within the renin–angiotensin system or alternative pathways contributing to hypertension. Fifthly, the paper fails to address the exclusion of unavailable studies (29 articles omitted due to unavailability), which may introduce selection bias affecting the thoroughness of the review. Sixthly, there is inadequate research on emergency reversibility. While the reversibility of siRNA-induced AGT suppression during emergencies is emphasized, the discussion lacks comprehensive support. Seventhly, most clinical outcomes rely on early-phase studies; therefore, the paper inadequately bridges the gap between preclinical success and real-world clinical applications. Eighthly, while the paper discusses reductions in blood pressure (e.g., declines in mmHg), there are limited quantitative comparisons between various RNA-based therapies and conventional treatments, raising concerns about feasibility. The article predominantly depends on data from specific population groups, raising concerns about the broader applicability of its conclusions to more diverse populations and offering minimal discussion of ethical concerns. 

## 5. Conclusions

RNAi-based therapies, particularly those using zilebesiran (a GalNAc-siRNA), have shown promising preclinical and clinical evidence for reducing BP and offer a novel therapeutic approach for patients with hypertension. Targeted delivery to hepatocytes using N-acetylgalactosamine conjugation enables effective and sustained AGT knockdown, with siRNAs demonstrating more consistent results than ASOs. These therapies present a potential solution to poor treatment adherence and efficacy issues associated with current antihypertensive drugs. However, challenges, such as genetic variability, long-term safety, and high costs, must be addressed. Future research should focus on confirming the efficacy and safety of RNAi in diverse patient populations and developing strategies to manage potential medical emergencies in patients undergoing RNAi therapy.

## Figures and Tables

**Table 1:** Characteristics of the studies included in this scoping review.

Title	Main Findings	Reference
Targeting angiotensinogen with N-acetylgalactosamine-conjugated small interfering RNA to reduce blood pressure	This manuscript explores the potential of targeting hepatic angiotensinogen (AGT) using N-acetylgalactosamine (GalNAc)-conjugated small interfering RNA (siRNA) as a novel therapeutic strategy for hypertension management. Current therapies face challenges due to poor adherence and RAS escape. GalNAc-siRNA effectively lowers blood pressure and provides renoprotective and cardioprotective benefits.	[8]
Zilebesiran, an RNA interference therapeutic agent for hypertension	Zilebesiran, an RNA interference therapeutic agent, is evaluated as an innovative treatment for hypertension. It targets hepatic angiotensinogen mRNA, reducing AGT production. The treatment significantly reduces serum AGT levels, reducing blood pressure up to 24 weeks. It is well-tolerated, offers potential benefits, and provides 24-h tonic control. Future research is needed to confirm its long-term efficacy and safety.	[13]
Emerging insights and future prospects for therapeutic application of siRNA targeting angiotensinogen in hypertension	Hypertension is a major cardiovascular risk factor, but less than half of treated patients are controlled due to nonadherence. Small interfering RNA therapies targeting hepatic angiotensinogen offer a precision medicine approach, with the potential for biannual dosing and 24-h tonic control.	[6]
A promising antihypertensive therapy inhibiting angiotensinogen synthesis	Zilebesiran, a siRNA targeting the liver, is a potential addition to the renin–angiotensin–aldosterone system-inhibiting drugs. It silences the AGT gene responsible for angiotensinogen synthesis, blocking the production of angiotensin II, a key factor in blood pressure elevation. Phase I clinical trials show a dose-dependent negative relationship with blood pressure/serum angiotensinogen levels, with sustained effects up to 6 months.	[14]
The emerging role of angiotensinogen in cardiovascular diseases	The potential of targeting hepatic AGT with GalNAc-siRNA as a novel and practical therapeutic approach for managing hypertension, with the added benefit of potentially improving medication adherence due to less frequent dosing requirements	[15]
RNA interference with zilebesiran for mild to moderate hypertension	A phase 2 trial found that subcutaneous doses of zilebesiran significantly decreased systolic blood pressure compared to a placebo. Nonserious adverse events occurred in 16.9% of patients, mainly injection site reactions and mild hyperkalemia. The study suggests zilebesiran could be an effective antihypertensive with quarterly or biannual dosing.	[16]
Nanoparticle-mediated RNA interference of angiotensinogen decreases blood pressure and improves myocardial remodeling in spontaneously hypertensive rats	The administration of small hairpin RNA (shRNA) targeting AGT significantly reduced hepatic AGT mRNA and protein levels, plasma AGT, and Ang II concentrations in SHRs. A single injection of AGT-shRNA nanoparticles resulted in a sustained reduction in systolic blood pressure by approximately 30 mmHg, lasting over 10 days. AGT silencing also improved cardiac hypertrophy, with reduced hypertrophy of cardiac muscle cells and improved myocardial ultrastructure. The study reported no significant side effects.	[17]
Targeting of hepatic angiotensinogen using chemically modified siRNAs results in significant and sustained blood pressure lowering in a rat model of hypertension	AGT-siRNA therapy significantly reduced hepatic AGT mRNA levels and plasma AGT concentrations without impairing liver function. A single intravenous injection lowered systolic BP in spontaneously hypertensive rats by 36 mmHg, with sustained effects lasting up to 7 days. The intervention targeted hepatic AGT mRNA without affecting AGT expression in extrahepatic tissues. AGT-siRNA treatment was safe and well-tolerated. Its potential for chronic BP management and adjunct therapy with standard RAAS inhibitors is also highlighted.	[18]
Conventional vasopressor and vasopressor-sparing strategies to counteract the blood pressure-lowering effect of small interfering RNA targeting angiotensinogen	Angiotensinogen siRNA, a gene targeted by hepatic angiotensinogen, significantly reduced plasma angiotensinogen concentrations by 99.2%, causing a 19 mmHg reduction in hypertensive rats. This reduction was reversed by angiotensin II or norepinephrine, and a gradual reversal was achieved with high sodium intake or fludrocortisone. The study found no significant renal dysfunction during siRNA treatment, but fludrocortisone administration led to weight loss and heart rate changes. This suggests the potential for the clinical implementation of angiotensinogen siRNA as a novel antihypertensive strategy.	[19]
Novel pharmacological approaches in the treatment of hypertension: a focus on RNA-based therapeutics	Zilebesiran reduced BP by up to 20 mm Hg and sustained it for 6 months after a single administration, likely due to its very effective knockdown effect on AGT. IONIS-AGT-LRx showed minor knockdown effects on AGT and marginal effects on BP.	[2]
Strong and sustained antihypertensive effect of small interfering RNA targeting liver angiotensinogen	The study demonstrates the effectiveness of siRNA in reducing mean arterial pressure (MAP) and enhancing cardioprotection. It found that siRNA monotherapy reduced cardiac hypertrophy markers and achieved the greatest reduction in MAP. The therapy also reduced hepatic AGT mRNA levels, lowering plasma AGT by 97.9%. It also significantly reduced renal and plasma angiotensin I levels. The therapy did not adversely affect renal function, and plasma potassium levels increased modestly. The study suggests that siRNA targeting hepatic AGT could enhance medication adherence. Further research is needed to explore safety, efficacy, and optimal dosing in clinical settings.	[10]
Novel antihypertensive agents for resistant hypertension: what does the future hold?	New therapeutic agents are being developed for better blood pressure control in people with resistant hypertension. These include mineralocorticoid receptor blockade, aminopeptidase A inhibitor, combined endothelin A and B receptor blocker, and aldosterone synthase inhibitor. These agents are in phase II development or phase III trials. Novel agents use antisense inhibition to block angiotensinogen development in the liver. Sacubitril/valsartan, initially developed as an antihypertensive, was approved as a heart failure agent.	[20]
Effect of angiotensinogen-targeted RNA interference in spontaneously hypertensive rats	Ad5-ACE-shRNA injection significantly reduced SBP and maintained antihypertensive effects for at least 14 days. ACE expressions decreased in Ad5-ACE-shRNA-treated SHRs compared to saline and Ad5 groups. LVW/BW ratio and myocardial collagen content were lower in Ad5-ACE-shRNA-treated SHRs but higher than in the WKY group. Myocardial ultrastructure was also significantly improved in Ad5-ACE-shRNA-treated SHRs.	[21]
Zilebesiran—the first siRNA-based drug in hypertensiology: why is it needed, and will it change the treatment approach for hypertension?	The results indicated that doses of 100 mg and 200 mg of zilebesiran significantly reduced serum AGT levels by over 90% after eight weeks, with an average reduction in systolic BP of 10 mmHg.	[22]
Angiotensinogen suppression: a new tool for treating cardiovascular and renal diseases	RNA-based therapies targeting the renin–angiotensin system (RAS) have shown promising results in suppressing angiotensin production, addressing the limitations of traditional RAS inhibitors. These therapies have shown significant reductions in blood pressure and have potential renoprotective effects, cardiovascular benefits, and metabolic syndrome benefits. Early-phase clinical trials show promising safety profiles and current clinical trials are continuing.	[23]

AGT, angiotensinogen; Ang II, angiotensin II; ASO, antisense oligonucleotide; BP, blood pressure; GalNAc, N-acetylgalactosamine; GalNAc-siRNA, N-acetylgalactosamine-conjugated siRNA; IONIS-AGT-LRx, Ionis Pharmaceuticals-angiotensinogen-ligand-conjugated antisense oligonucleotide; KARDIA-1, a clinical study conducted by Ionis Pharmaceuticals; mRNA, messenger RNA; RAAS, renin–angiotensin–aldosterone system; RAS, renin–angiotensin system; RNA, ribonucleic acid; SBP, systolic blood pressure; SHRs, spontaneously hypertensive rats; siRNA, small interfering RNA; WKY, Wistar Kyoto rats.

**Table 3:** Clinical trials with RNAi for treating hypertension.

Drug	Clinical Trial Identifier	Objective	Phase
GalNAc-siRNA (ALN-AGT)	NCT04936035 (KARDIA-1)	To evaluate the drug effects on SBP and DBP and characterize its pharmacodynamic effects.	Phase 2
GalNAc-siRNA (ALN-AGT)	NCT03934307	To evaluate the drug’s safety, tolerability, PK, and pharmacodynamics.	Phase 1
GalNAc-siRNA (ALN-AGT)	NCT05103332 (KARDIA-2)	To evaluate the effects of zilebesiran as adjunct therapy on SBP and DBP.	Phase 2
GalNAc-siRNA (ALN-AGT)	NCT06272487 (KARDIA-3)	To evaluate zilebesiran as adjunct therapy in high-risk patients.	Phase 2
GalNAc-ASO (IONIS-AGT-LRx)	NCT04714320 (ASTRAAS)	To evaluate the drug effects vs. those of a placebo on SBP, DBP, and plasma AGT.	Phase 2
GalNAc-ASO (IONIS-AGT-LRx)	NCT03714776	Phase 2, randomized, double-blind, placebo-controlled study in mild hypertensive participants.	Phase 2
GalNAc-ASO (IONIS-AGT-LRx)	NCT04083222	To evaluate the drug effects on AGT and SBP in uncontrolled hypertensive participants.	Phase 2

**Table 4:** Preclinical stages for hypertension applications.

Feature	Antisense Oligonucleotides (ASO	RNA Interference (RNAi)
Mechanism of Action	Binds to angiotensinogen (AGT) mRNA to degrade or block translation, reducing AGT protein levels	Silences angiotensinogen gene expression by targeting AGT mRNA through RISC-mediated degradation
Primary Target	Angiotensinogen (AGT), a precursor in the renin–angiotensin system (RAS)	Angiotensinogen (AGT) to disrupt RAS function
Therapeutic Goal	Decrease AGT levels to reduce blood pressure	Long-term suppression of AGT expression to achieve sustained blood pressure control
Delivery Method	Typically subcutaneous injections	Lipid nanoparticle delivery systems or conjugation with targeted molecules
Duration of Effect	Requires more frequent dosing (e.g., weekly or biweekly)	Longer-lasting effects due to stable incorporation into RISC complexes
Clinical Trials	Emerging studies with promising results in reducing blood pressure	Some RNAi therapies (e.g., inclisiran for other indications) show potential for hypertension
Specificity for Hypertension	Focused on AGT as a critical regulator of blood pressure	Potential to target multiple pathways within the renin–angiotensin–aldosterone system
Challenges	Risk of off-target effects and immune responses	Delivery system complexity and cost remain significant hurdles
Advantages	Simpler synthesis and easier scalability for production	Sustained gene silencing offers potential for long-term blood pressure management
Current Status	Preclinical and early clinical trials	Preclinical stages for hypertension applications

## Data Availability

Research data supporting this publication are available upon request from the corresponding author.
